# The copper-responsive regulator CsoR is indirectly involved in *Bradyrhizobium diazoefficiens* denitrification

**DOI:** 10.1093/femsle/fnad084

**Published:** 2023-08-12

**Authors:** Pedro J Pacheco, Juan J Cabrera, Andrea Jiménez-Leiva, María J Torres, Andrew J Gates, Eulogio J Bedmar, David J Richardson, Socorro Mesa, Germán Tortosa, María J Delgado

**Affiliations:** Department of Soil and Plant Microbiology, Estación Experimental del Zaidín, CSIC, C/Profesor Albareda 1, E-18008 Granada, Spain; Department of Soil and Plant Microbiology, Estación Experimental del Zaidín, CSIC, C/Profesor Albareda 1, E-18008 Granada, Spain; Department of Soil and Plant Microbiology, Estación Experimental del Zaidín, CSIC, C/Profesor Albareda 1, E-18008 Granada, Spain; School of Biological Sciences, University of East Anglia, Norwich Research Park, Colney Lane, Norwich NR4 7TJ, United Kingdom; Department of Biochemistry and Molecular Biology, Campus Universitario de Rabanales, University of Córdoba, Ed. C6, Planta Baja, 14071 Córdoba, Spain; School of Biological Sciences, University of East Anglia, Norwich Research Park, Colney Lane, Norwich NR4 7TJ, United Kingdom; Department of Soil and Plant Microbiology, Estación Experimental del Zaidín, CSIC, C/Profesor Albareda 1, E-18008 Granada, Spain; School of Biological Sciences, University of East Anglia, Norwich Research Park, Colney Lane, Norwich NR4 7TJ, United Kingdom; Department of Soil and Plant Microbiology, Estación Experimental del Zaidín, CSIC, C/Profesor Albareda 1, E-18008 Granada, Spain; Department of Soil and Plant Microbiology, Estación Experimental del Zaidín, CSIC, C/Profesor Albareda 1, E-18008 Granada, Spain; Department of Soil and Plant Microbiology, Estación Experimental del Zaidín, CSIC, C/Profesor Albareda 1, E-18008 Granada, Spain

**Keywords:** Cu-responsive regulator, gene expression, nitrite reductase, nitric oxide reductase

## Abstract

The soybean endosymbiont *Bradyrhizobium diazoefficiens* harbours the complete denitrification pathway that is catalysed by a periplasmic nitrate reductase (Nap), a copper (Cu)-containing nitrite reductase (NirK), a *c*-type nitric oxide reductase (cNor), and a nitrous oxide reductase (Nos), encoded by the *napEDABC, nirK, norCBQD*, and *nosRZDFYLX* genes, respectively. Induction of denitrification genes requires low oxygen and nitric oxide, both signals integrated into a complex regulatory network comprised by two interconnected cascades, FixLJ–FixK_2_–NnrR and RegSR–NifA. Copper is a cofactor of NirK and Nos, but it has also a role in denitrification gene expression and protein synthesis. In fact, Cu limitation triggers a substantial down-regulation of *nirK, norCBQD*, and *nosRZDFYLX* gene expression under denitrifying conditions. *Bradyrhizobium diazoefficiens* genome possesses a gene predicted to encode a Cu-responsive repressor of the CsoR family, which is located adjacent to *copA*, a gene encoding a putative Cu^+^-ATPase transporter. To investigate the role of CsoR in the control of denitrification gene expression in response to Cu, a *csoR* deletion mutant was constructed in this work. Mutation of *csoR* did not affect the capacity of *B. diazoefficiens* to grow under denitrifying conditions. However, by using qRT-PCR analyses, we showed that *nirK* and *norCBQD* expression was much lower in the *csoR* mutant compared to wild-type levels under Cu-limiting denitrifying conditions. On the contrary, *copA* expression was significantly increased in the *csoR* mutant. The results obtained suggest that CsoR acts as a repressor of *copA*. Under Cu limitation, CsoR has also an indirect role in the expression of *nirK* and *norCBQD* genes.

## Teaser

Identification of a new role of the Cu-responsive regulator CsoR in the control of denitrification in *Bradyrhizobium diazoefficiens*.

## Introduction

Rhizobia are soil bacteria with the unique ability to establish symbiosis with their host legume through the formation of nodules in their roots, where they differentiate into bacteroids, which are able to synthesize the enzyme responsible for the symbiotic dinitrogen (N_2_) fixation, the nitrogenase (Mahmud et al. 2020). Given the sensitivity of this enzyme to oxygen (O_2_), the steady-state concentration within root nodules is typically in the tens of nanomolar, approximately four orders of magnitude lower than equilibrium levels in the external environment (Udvardi and Poole 2013). Under certain conditions, such as flooding or nitrate (NO_3_^−^) excess in the rhizosphere, bacteroids can make use of other inorganic terminal electron acceptors such as NO_3_^−^ or nitrite (NO_2_^−^), which are reduced to N_2_ through the denitrification pathway, producing nitric oxide (NO) and nitrous oxide (N_2_O) as intermediates (reviewed by Salas et al. 2021). At the cellular level, NO acts as a key signal molecule at low concentrations, but is a potent cytotoxic compound at high concentrations. N_2_O is the dominant nitrogenous greenhouse gas in the atmosphere that has a warming capacity 296 times greater than that of CO_2_ due to its radiative capacity, and is responsible for 6% of current global warming. Because of its long atmospheric lifetime (120–150 years), it can be converted by photolysis in the stratosphere into NO, causing ozone layer depletion (Erisman et al. 2015).

*Bradyrhizobium diazoefficiens*, the soybean endosymbiont, is considered as a model to study rhizobial denitrification because, in addition to fix N_2_, is the only species able to grow under anoxic conditions with NO_3_^−^ as sole nitrogen source by the complete denitrification pathway, which has been deeply characterized under both free-living and symbiotic conditions (reviewed by Salas et al. 2021). This bacterium possesses the whole set of *napEDABC, nirK, norCBQD*, and *nosRZDFYLX* denitrification genes, which encode a periplasmic nitrate reductase system (Nap), a Cu-containing nitrite reductase (NirK), a *c*-type nitric oxide reductase system (cNor), and a nitrous oxide reductase (Nos), respectively. As in many other denitrifiers, the expression of these genes in *B. diazoefficiens* requires O_2_ limitation and the presence of NO_3_^−^ or another N oxide derived from its reduction (reviewed by Torres et al. 2016, Salas et al. 2021).

**Figure 1.**
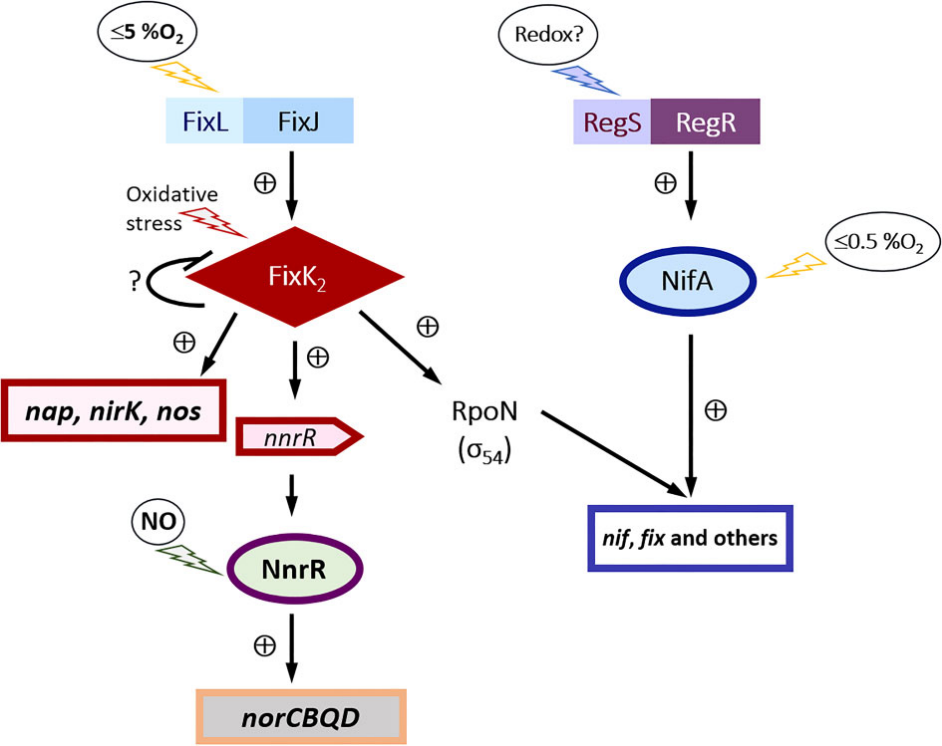
Regulatory network of denitrification genes in *B. diazoefficiens* in response to O_2_ and NO. The regulatory protein FixK_2_ is the direct activator of *nap, nirK*, and *nos* genes in response to low O_2_ conditions (≤ 5% O_2_). This regulator also activates *nnrR*, which encodes the transcription factor NnrR, necessary for induction of *nor* genes in response to NO. FixK_2_ is also the link to the RegSR–NifA cascade responsible for activating the *nif* and *fix* genes in response to very low O2 conditions (≤ 0.5% O_2_).

In this context, this bacterium detects the low O_2_ signal via two interconnected regulatory cascades: FixLJ–FixK_2_–NnrR and RegSR–NifA (reviewed by Bueno et al. 2012, Torres et al. 2016, Salas et al. 2021) (Fig. 1). When a moderate decrease in O_2_ (≤ 5% in the gas phase) occurs, the two-component regulatory system, FixLJ, activates *fixK*_2_ transcription (Sciotti et al. 2003). Then, FixK_2_ protein induces *nap, nirK*, and *nos* gene expression, as well as *rpoN*_1_ and *nnrR* genes among others (Mesa et al. 2008, Bueno et al. 2017, Torres et al. 2017). The product of the latter gene, NnrR, is the direct regulator of the *norCBQD* genes in response to NO (Bueno et al. 2017, Jiménez-Leiva et al. 2019). The second O_2_-responsive regulatory cascade, RegSR–NifA, responds to lower O_2_ concentrations. In this cascade, the two-component regulatory system RegSR induces the expression of *nifA*. When O_2_ concentration substantially decreases (≤ 0.5% in the gas phase), NifA induces the expression of nitrogen-fixation genes (*nif* and *fix*), among others (Fig. 1).

Besides O_2_ and NO, copper (Cu) is an emerging candidate in the regulation of denitrification, since it is an essential cofactor in two enzymes involved in this process, NirK and NosZ. Pacheco et al. (2022) recently proposed that Cu represents an essential factor not only for denitrifying enzymatic activities, but also for the regulation of gene expression, as well as protein synthesis and maturation. Particularly, the expression of *nirK, nor*, and *nos* genes, but not *nap* genes diminished under Cu-limitation in cells grown under NO_3_^−^-amended O_2_-limiting conditions, which suggests the involvement of a specific Cu-responsive regulator in this control. In bacteria, various types of Cu-sensing transcriptional regulators have been characterized, such as CopY in *Escherichia coli* and *Enterococcus hirae* or CsoR in *Mycobacterium tuberculosis* (reviewed by Rademacher and Masepohl 2012). In the latter, the *csoR* gene constitutes an operon together with other two genes: *rv0968*, encoding a hypothetical protein annotated as DUF1490, and *rv0969* (*ctpV*), which codes for a Cu^+^-ATPase transporter denoted as CtpV, presumably involved in Cu excretion (Liu et al. 2007). CsoR and CtpV homologs have also been described in the rhizobial species *Bradyrhizobium liaoningense* CCNWSX0360, where their involvement in heavy metal-response regulation has been suggested. In this bacterium, CtpV is denoted as CopA (Liang et al. 2016).

In the present work, a gene encoding a protein from the CsoR family of regulators and another responsible for the synthesis of the Cu^+^-ATPase transporter, CopA, were unveiled in the *B. diazoefficiens* genome. To investigate the possible involvement of CsoR in the regulation of denitrification, a mutant strain was constructed by deletion of the *csoR* gene. This strain was further used in growth and gene expression analyses. The results obtained suggest that CsoR is not essential for bacterial growth, but may play an indirect role in the Cu-mediated control of *nirK* and *norC* expression. Therefore, this work assesses a novel role of CsoR in *B. diazoefficiens* denitrification.

## Material and methods

### Bacterial strains and growth conditions

All the strains used in this work are listed in Table 1. To generate the *csoR* deletion mutant, upstream (600 bp) and downstream (480 bp) DNA fragments flanking Bdiaspc4_RS03270 were amplified by PCR using *csoR*_Up_For_*Xba*I/*csoR*_Up_Rev_*Bam*HI and *csoR*_Down_For_*Bam*HI/*csoR*_Down_Rev_*Eco*RI primer pairs (Table S1, Supporting Information). The 1080-bp fragment was inserted into pK18*mobsacB* (Schäfer et al. 1994), yielding plasmid pDB4030. After corroborating the integrity of the cloned fragment by sequencing using the primers pSRKC1_F, pK18_4, and the internal primer *csoR*_IN_For (Table S1, Supporting Information), pDB4030 was conjugated with *B. diazoefficiens* 110*spc*4 obtaining the markerless *csoR* deletion mutant strain Bd4030 (denoted as Δ*csoR* throughout the manuscript) after a double recombination event.

**Table 1.** Strains used in this work.

Strain	Relevant characteristics	References
*E. coli*		
DH5α	*supE44*, Δ*lacU169*, (Φ80 *lac*ZΔM15), 5*hsdR17, recA1, endA1, gyrA96, thi*-*1, relA1*	Sambrook et al. (1989)
S17.1	Sm^r^ Spc^r^ Tp^r^; *thi, pro, recA, hsdR, hsdM*, RP4Tc::Mu, Km::Tn7	Simon et al. (1983)
*B. diazoefficiens*		
110*spc*4	Cm^r^ Spc^r^, WT	Regensburger and Hennecke (1983)
4030	Cm^r^ Spc^r^; markerless *csoR* deletion mutant strain derived from 110*spc*4	This work
110*spc*4-BG0614	Cm^r^ Spc^r^ Tc^r^; 110*spc*4::P*napE-lacZ*	This work
110*spc*4-RJ2498	Cm^r^ Spc^r^ Tc^r^; 110*spc*4::P*nirK-lacZ*	Mesa et al. (2003)
110*spc*4-RJ2499	Cm^r^ Spc^r^ Tc^r^; 110*spc*4-P*norC-lacZ*	Mesa et al. (2003)
110*spc*4-BG0301	Cm^r^ Spc^r^ Tc^r^; 110*spc*4::P*nosR-lacZ*	Torres et al. (2017)
4030-BG0614	Cm^r^ Spc^r^ Tc^r^; 4030::P*napE-lacZ*	This work
4030-RJ2498	Cm^r^ Spc^r^ Tc^r^; 4030::P*nirK-lacZ*	This work
4030-RJ2499	Cm^r^ Spc^r^ Tc^r^; 4030::P*norC-lacZ*	This work
4030-BG0302	Cm^r^ Spc^r^ Tc^r^; 4030::P*nosR-lacZ*	This work

*Escherichia coli* cells were cultured in Luria–Bertani medium (Miller 1972) at 37°C. The following antibiotics were added to the medium at the following concentrations ($\mu$g ml^−1^): streptomycin, 25; spectinomycin, 25; kanamycin, 25; and tetracycline, 10.

*Bradyrhizobium diazoefficiens* cells were grown routinely under oxic conditions at 30°C in PSY complete medium (Mesa et al. 2008) to obtain cellular mass. Subsequent experiments under microoxic conditions were carried out using a vitamin-free modified Vincent’s minimal medium (BVM; Vincent 1970, Becker et al. 2004) amended with 10 mM KNO_3_ (BVMN). This medium was supplemented (per litre) with 20 mM l-arabinose and 1 ml from a mineral solution originally developed by Bishop et al. (1976). Final pH was adjusted around 6.8 with 2 M NH_3_.

The specific Cu concentrations assayed in this study are extensively described in Pacheco et al. (2022). Briefly, the standard Cu concentration of the BVMN medium (0.02 $\mu$M) is denoted as Cu-S. A concentration of 13 $\mu$M is referred in this manuscript as high Cu, denoted as Cu-H. Finally, the Cu-limiting medium (denoted as Cu-L) was achieved by omitting CuSO_4_·5H_2_O from the mineral solution, and by adding 1 mM l-ascorbate (Cu(II) reducing agent) and 10 $\mu$M bathocuproine disulfonic acid (BCS) [Cu(I) chelating agent] in order to decrease Cu availability. In the case of Cu-L medium, glassware was treated with 0.1 M HCl and rinsed afterwards with double-distilled water (Serventi et al. 2012).

For microoxic conditions, 17-ml rubber-stoppered tubes or 250 or 500-ml rubber-stoppered flasks were filled with 3 ml, 5 ml, or 100 ml, respectively, of BVMN (1:5 ratio between air and liquid phases). Then, these tubes or flasks were flushed at the starting point of the incubation with a gas mixture consisting of 2% (v/v) O_2_ and 98% (v/v) N_2_, and were incubated at 30°C with agitation at 170 rpm.

Antibiotics used in *B. diazoefficiens* cultures were provided at the following concentrations ($\mu$g ml^−1^): spectinomycin, 200 (solid), 100 (liquid); kanamycin, 200 (solid), 100 (liquid); and tetracycline, 50 (solid), 25 (liquid).

### Determination of β-galactosidase activity and protein concentration

β-Galactosidase activity levels were analysed by using permeabilized cells from at least three independent cultures, assayed in triplicate for each strain and condition, as described by Cabrera et al. (2016). Specific activity was calculated in Miller Units (Miller 1972). Protein concentration was estimated by using the Bradford reagent (Bio-Rad, CA, USA) (Bradford 1976).

### Intracellular copper determination

Cu concentration was analysed in the cells using the Inductively Coupled Plasma Optical Emission Spectrometer (ICP-OES) available at the Instrumental Technical Service of EEZ (Granada, Spain). Before measurements, cell samples were mineralized by microwave digestion in the presence of the acidic HCl-HF (1/1, v/v) mixture. Data were expressed as $\mu$g Cu mg protein^−1^.

### Quantitative real-time PCR analysis

*Bradyrhizobium diazoefficiens* cells grown for 48 h under microoxic conditions in Cu-L, Cu-S, or Cu-H BVMN medium were harvested. Then, total RNA isolation and cDNA synthesis were performed according to Hauser et al. (2007), Mesa et al. (2008), and Lindemann et al. (2007). The subsequent analysis of *napE, nirK, norC*, and *nosR* expression was carried out by qRT-PCR using a QuantStudio 3 Real-Time PCR system (Thermo-Fisher Scientific, MA, USA). Primers for the PCR reactions were previously designed (Table S1, Supporting Information). Each PCR reaction contained 9.5 $\mu$l of iQTM SYBR Green Supermix (Bio-Rad), 2 mM (final concentration) of individual primers, and appropriate dilutions of different cDNA samples, obtaining a total volume of 19 $\mu$l per well. Reactions were run in triplicate and melting curves were generated in order to verify the specificity of each amplification. Finally, relative changes in gene expression were calculated according to the method described by Pfaffl (2001). 16S *rrn* expression was used as reference for normalization (see primers in Table S1, Supporting Information).

### Statistical analysis

The total number of replicates appears in each Figure. Inferential statistics were performed by the application of a parametric ANOVA for unpaired treatments. Next, a *post hoc* Tukey HSD test at *P* ≤ .05 with SPSS (v27) software was performed.

## Results and discussion

### Identification of a putative *csoR* gene in *B. diazoefficiens*

**Figure 2.**
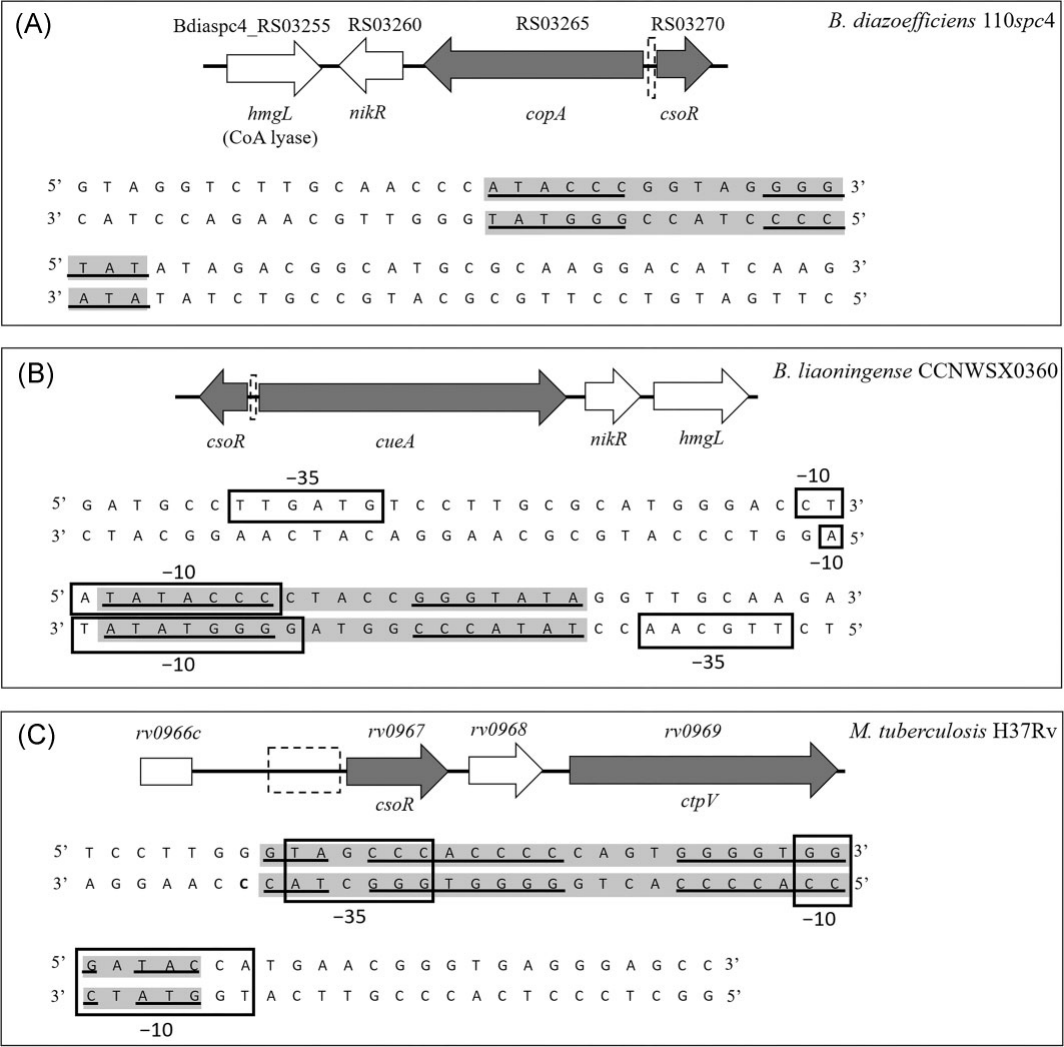
Genomic context of the *csoR* gene in *B. diazoefficiens* 110*spc*4 (A), *B. liaoningense* CCNWSX0360 (B), and *M. tuberculosis* H37v (C). The nucleotide sequence of the regions marked with a dash square adjacent to *csoR* are also shown. In each sequence, the CsoR boxes are shaded in grey, with the inverted repeats underlined. In (B) and (C), the −10 and −35 regions of the *csoR* promoter are indicated with an open black box. (B) and (C) are adapted from Liang et al. (2016) and Liu et al. (2007), respectively.

The analysis of *B. diazoefficiens* 110*spc*4 genome unveiled a small open reading frame (ORF) annotated as Bdiaspc4_RS03270, encoding a CsoR-like Cu-sensing transcriptional repressor. This ORF is inversely oriented and located immediately adjacent to *copA* (Bdiaspc4_RS03265), a gene encoding a P-type Cu^+^ transporter (Fig. 2A). In the 3′ end of *copA*, we found *nikR*, which putatively encodes a nickel (Ni) transporter, and *hmgL*, encoding a possible hydroxymethylglutaryl-CoA lyase (Fig. 2A). This genetic arrangement is similar to that observed in *B. liaoningense* CCNWSX0360 (Fig. 2B) (Liang et al. 2016), and *M. tuberculosis* H37Rv (Fig. 2C) (Liu et al. 2007). As shown in Fig. 2(B) and (C), a (G + C)-rich pseudo-palindromic sequence of about 19 bp in *B. liaoningense* and 28 bp in *M. tuberculosis* was identified within the promoter region of *csoR*. These sequences correspond presumably to the CsoR-binding site, which has been called the CsoR box. In both species, this box encompasses inverted repeats coinciding with the −35 or −10 promoter elements (Fig. 2B and C). The 6-bp inverted repetitions shown in *B. diazoefficiens* (Fig. 2A) suggest the presence of a putative 17-bp CsoR box.

By using KEGG database, we found that Bdiaspc4_RS03270 encodes a protein of 91 amino acids. The deduced amino acid sequence of this protein was aligned against other proteins from the CsoR family (Figure S1, Supporting Information) annotated from other denitrifying species such as *Bacillus subtilis, B. diazoefficiens* USDA110, *B. japonicum, B. liaoningense, E. coli, Paracoccus denitrificans, Paracoccus pantotrophus, Pseudomonas aeruginosa, Pseudomonas fluorescens*, and *Rhodopseudomonas palustris*. The sequences of the characterized CsoR proteins from *Corynebacterium glutamicum* (Teramoto et al. 2012), *M. tuberculosis* (Liu et al. 2007), and *Thermus thermophilus* (Sakamoto et al. 2010) were also included. The CsoR sequence alignment for *B. diazoefficiens* 110*spc*4 showed 100%, 97.80%, 96.70%, and 91.21% identity with the CsoR-like protein sequence from *B. diazoefficiens* USDA110, *B. japonicum, B. liaoningense*, and *R. palustris*, respectively (Table S2, Supporting Information), suggesting that these proteins are orthologs and that the CsoR sequence from *B. diazoefficiens* is absolutely conserved between 110*spc*4 and USDA110 strains. Moreover, the sequence of *B. diazoefficiens* CsoR shared identity with the sequences from other species including some denitrifiers (Figure S1 and Table S2, Supporting Information). The predicted structure of the *B. diazoefficiens* CsoR by AlphaFold (neurosnap.ai, 15/07/2023) as homodimer, showed high similarity with that from *M. tuberculosis* and *T. thermophilus* solved by Liu et al. (2007) and Sakamoto et al. (2010), respectively. The model indicates the presence of three α-helices per monomer, with compatible Cu(I) binding sites (C-H-C motif) predicted between C33 of one monomer and H58 and C62 from the other (Figure S2, Supporting Information). These residues are conserved in the majority of the sequences analysed in Figure S1 (Supporting Information) , except for *E. coli*, which displays a C-H-R motif, and *T. thermophilus* and *P. pantotrophus*, with C-H-H motifs (Figure S1, Supporting Information). Moreover, R12, Y32, R49, and E81 proposed in other CsoR proteins necessary for protein binding to DNA (Liu et al. 2007) are conserved in *B. diazoefficiens* (Figure S1, Supporting Information). CsoR ortologous have been resolved as homodimers and homotetramers (Liu et al. 2007, Sakamoto et al. 2010). Other highly conserved residues (Figure S1, Supporting Information) could be presumably involved in the interaction between CsoR monomers to originate the oligomer. Considering these results, Bdiaspc4_RS03270 has been denoted as the *csoR* gene henceforth in this manuscript.

### Growth and gene expression analyses in a Δ*csoR* strain

**Figure 3.**
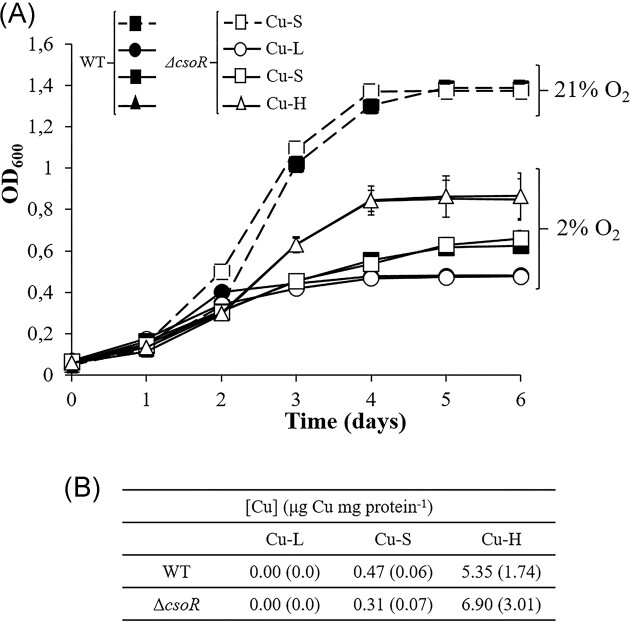
(A) Growth of *B. diazoefficiens* 110*spc*4 (black symbols) and Δ*csoR* strain (open symbols) under Cu limitation (Cu-L, i.e. chelated) (circles), standard Cu (Cu-S, 0.02 $\mu$M) (squares), or high Cu (Cu-H, 13 $\mu$M) (triangles) BVMN media under oxic (dash line, 21% O_2_) or microoxic conditions (solid line, 2% O_2_). Data are means with standard error bars from at least two independent cultures assayed in triplicate, and where not visible, these were smaller than the symbols. (B) Intracellular Cu concentration of *B. diazoefficiens* 110*spc*4 and Δ*csoR* mutant grown for 3 days under microoxic conditions in Cu-L, Cu-S, or Cu-H BVMN media. Data expressed as $\mu$g Cu mg protein^−1^, are means with standard deviation from at least two independent cultures assayed in triplicate.

In order to investigate the possible role of CsoR in denitrification gene expression under Cu limitation, a *B. diazoefficiens* markerless deletion mutant in the *csoR* gene (denoted as Δ*csoR*) was constructed (see the section ‘Material and Methods’). Growth of *B. diazoefficiens* 110*spc*4 (WT) and the *csoR* mutant was monitored throughout an incubation period of 6 days under microoxic conditions in Cu limitation (i.e. chelated, Cu-L), standard Cu (0.02 $\mu$M, Cu-S), or high Cu (13 $\mu$M, Cu-H) BVMN media. As Cu concentration did not affect WT growth under oxic conditions (Pacheco et al. 2022), Cu-S oxic growth of each strain was used as control in our experiments. It is important to note that, as shown in Fig. 3(A), Cu-H (13 $\mu$M) is not actually toxic for *B. diazoefficiens* growth. In fact, Cu-H improves the growth rate compared to Cu-S (Fig. 3A), as previously demonstrated (Pacheco et al. 2022). When *B. diazoefficiens* cells were grown under different Cu concentrations ranging from 13 $\mu$M to 10 mM Cu, the growth profile obtained with 1 mM was similar to that observed with Cu-S. However, 2 and 3 mM Cu triggered a significant delay in growth that was completely abolished in the presence of 10 mM (data not shown). Hence, Cu-H (13 $\mu$M) is simply a Cu concentration above the standard Cu level for BVMN medium (Cu-S, 0.02 $\mu$M), i.e. suitable for our research purposes in the present work.

As shown in Fig. 3(A), under microoxic conditions, no differences in growth behaviour were observed in the Δ*csoR* in comparison to the WT, independently of the Cu concentration, i.e. growth was significantly affected in both strains by Cu limitation, compared to Cu-S or Cu-H grown cells. Consequently, CsoR is not apparently involved in *B. diazoefficiens* growth under microoxic conditions in response to Cu. Analyses of Cu concentration in cells revealed significant differences of Cu levels between cells grown in Cu-L, Cu-S, or Cu-H media during 3-days under microoxic conditions (Fig. 3B). As expected, Cu could not be detected in Cu-L grown cells from any of the strains. However, Cu levels increased to about 0.5 and 5 $\mu$g Cu mg protein^−1^ in WT Cu-S and Cu-H grown cells, respectively (Fig. 3B). Interestingly, under Cu-S conditions, Cu concentration in the *csoR* mutant was significantly lower than that from WT cells (0.31 $\mu$g Cu mg protein^−1^ versus 0.47 $\mu$g Cu mg protein^−1^, respectively). In contrast, no significant differences in Cu levels were observed in Cu-H grown WT cells compared to the *csoR* mutant (Fig. 3B).

**Figure 4.**
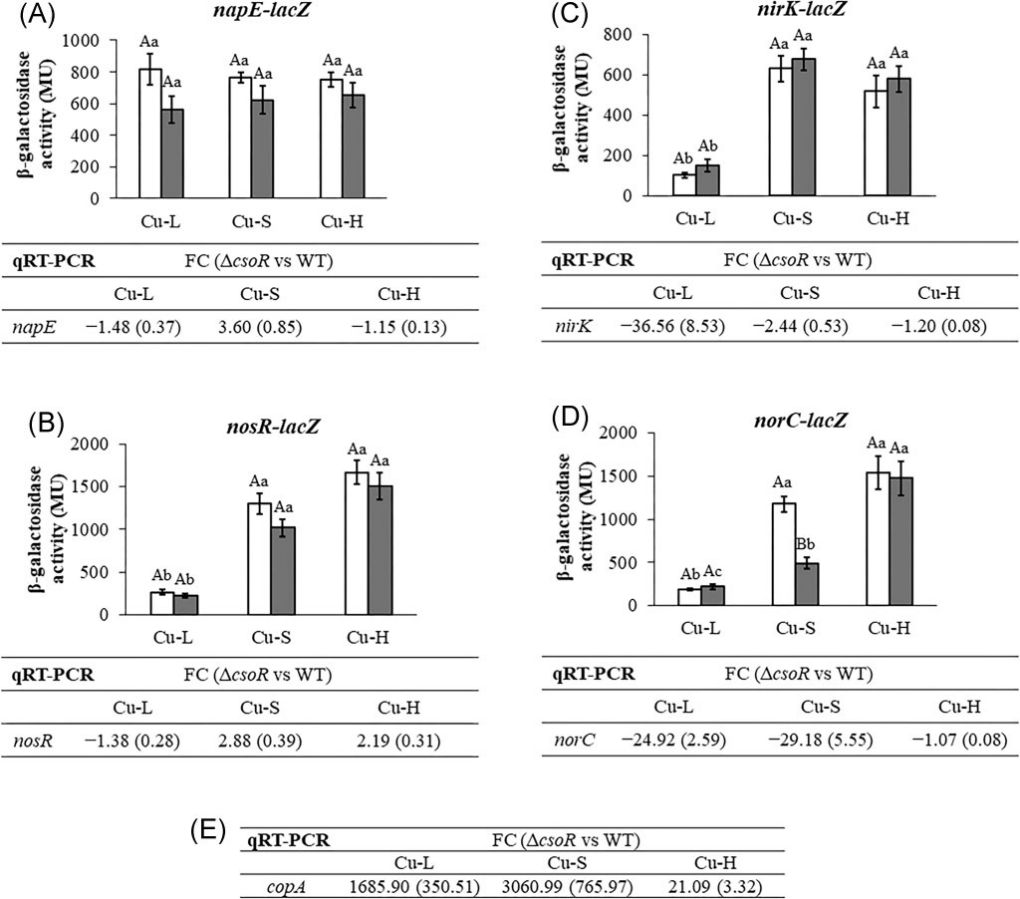
Transcriptional expression of denitrification genes monitored as β-galactosidase activity (histograms) from *napE-lacZ* (A), *nosR-lacZ* (B), *nirK-lacZ* (C), and *norC-lacZ* (D) transcriptional fusions chromosomally integrated in *B. diazoefficiens* 110*spc*4 (white bars) or Δ*csoR* strain (grey bars) grown microaerobically in Cu-L, Cu-S, or Cu-H BVMN media for 3 days. Data expressed as MU are means with standard deviation bars from at least three independent cultures assayed in triplicate. Same lower- or upper-case letters in each figure indicate that values are not statistically different according to a *post hoc* Tukey HSD test at *P* ≤ .05; lower-case letters indicate comparisons between Cu conditions, while upper-case letters mean comparisons between strains. Below each histogram, qRT-PCR expression of each denitrification gene, *napE* (A), *nosR* (B), *nirK* (C), and *norC* (D), is shown. (E) qRT-PCR values of *copA* gene in Cu-L, Cu-S, or Cu-H BVMN media. Fold-change (FC) values refer to differences in expression in the Δ*csoR* mutant relative to the WT. Data are means with standard deviation in parentheses from at least three independent cultures assayed in triplicate.

Next, the influence of *csoR* mutation on denitrification gene expression was determined by measuring β-galactosidase activity of transcriptional *lacZ* fusions and performing qRT-PCR assays (Fig. 4). For the former approach, Δ*csoR* strain derivatives harbouring *napE-lacZ, nirK-lacZ, norC-lacZ*, or *nosR-lacZ* transcriptional fusions were firstly constructed (see Table 1). Then, both WT and Δ*csoR* cells were grown microoxically in Cu-L, Cu-S, or Cu-H BVMN media. While β-galactosidase activity was measured after 72 h of incubation, when the maximal expression levels of these fusions is reached (Pacheco et al. 2022), for qRT-PCR experiments cells were collected after 48 h.

As shown in Fig. 4(A), both strains, WT and Δ*csoR*, displayed similar expression levels for *napE–lacZ* fusion independently of the Cu condition assayed (*P* > .05). Regarding *nosR–lacZ* expression, as previously observed by Pacheco et al. (2022), β-galactosidase activity was significantly reduced in Cu-L comparing to Cu-S WT cultures (Fig. 4B). However, no significant differences were observed between WT and Δ*csoR* strains regardless of the Cu concentration (*P* > .05) (Fig. 4B). These results were confirmed by qRT-PCR (Fig. 4A and B), indicating that *nap* and *nos* gene expression was not affected by *csoR* deletion.

Regarding *nirK–lacZ* and *norC–lacZ* fusions, no differences were observed between strains in Cu-L medium (*P* > .05) (Fig. 4C and D). However, the results obtained in qRT-PCR assays showed that *nirK* and *norC* expression levels were ~37-fold and 25-fold lower, respectively, in Δ*csoR* compared to WT under Cu limitation (Fig. 4C and D). This might be an indication of a possible post-transcriptional control in the Δ*csoR* mutant that affects *nirK* and *norC* mRNA stability.

Under Cu-S conditions, *nirK–lacZ* expression between strains was not significantly different (*P* > .05) (Fig. 4C), and this result was confirmed by qRT-PCR, since *nirK* expression analyses did not show a relevant change in the WT compared to Δ*csoR* (Fig. 4C). Concerning *norC–lacZ* expression in Cu-S medium, lower levels (about 3-fold less) were observed in the *csoR* mutant compared to those found for the WT strain (*P* < .05) (Fig. 4D). Similarly, *norC* expression levels obtained by qRT-PCR in Cu-S were ~29-fold lower in Δ*csoR* compared to WT (Fig. 4D). This significant decrease suggests that CsoR might be relevant to induce maximal *nor* genes expression in response to standard Cu levels (0.02 $\mu$M) in the growth medium.

In Cu-H medium (13 $\mu$M Cu), there were no significant differences in expression for any of the transcriptional fusions assayed (*napE, nirK, norC*, or *nosR–lacZ*), and these results were confirmed by qRT-PCR (Fig. 4A–D), indicating that CsoR may be not relevant for *B. diazoefficiens* denitrification gene induction under this Cu condition.

**Figure 5.**
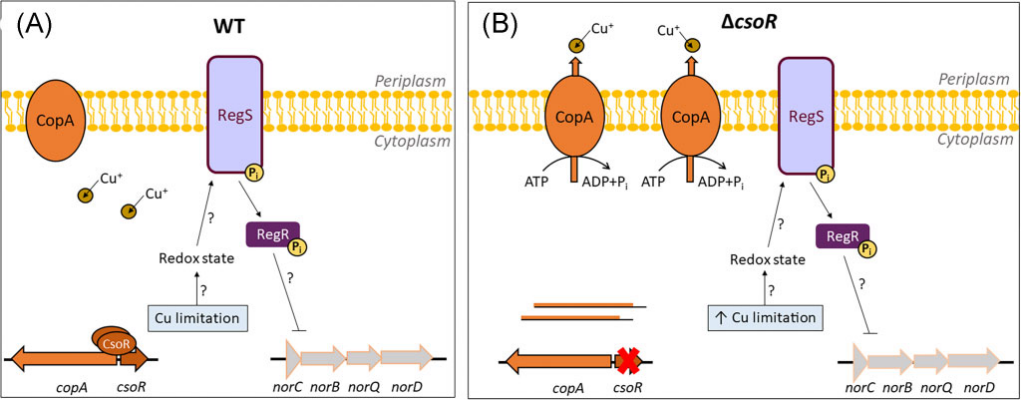
Schematic representation of the possible events occurring inside the cell under Cu limitation in the WT (A) or Δ*csoR* (B) grown under microoxic (2% O_2_) conditions. In the WT grown under Cu limitation (A), CsoR remains attached to the *csoR–copA* divergon, preventing *copA* maximal transcription levels. The synthesized P-type ATPase CopA proteins would be inactive because of the low cytosolic Cu availability. In Δ*csoR* grown under Cu limitation (B), CsoR is absent and *copA* expression would be derepressed making the cytosolic Cu concentration even more limiting (expressed in the figure as ↑ Cu-limitation). The results obtained in this work suggest that RegR could be involved in the control of *nor* genes under Cu limitation. Question marks indicate nondemonstrated events. Perpendicular lines indicate negative control.

Next, we investigated the possible involvement of CsoR in *copA* control. Therefore, we performed qRT-PCR experiments to analyse *copA* expression in the WT and Δ*csoR* strains grown under the battery of Cu concentrations assayed in this study. As shown in Fig. 4(E), *copA* expression was about 1700-fold and 3100-fold higher in the Δ*csoR* strain compared to the WT, when cells were grown under Cu-L or Cu-S conditions, respectively. These results suggest that CsoR could be involved in Cu response regulation as a repressor of *copA* either in Cu-L or Cu-S. When Cu concentration increased up to 13 $\mu$M (denoted as Cu-H in this work), *copA* expression was only about 21-fold higher in the *csoR* mutant compared to the WT (Fig. 4E). This result suggests, as previously demonstrated in *M. tuberculosis* (Liu et al. 2007), that CsoR might be attached to the promoter region under low Cu availability, thus causing *csoR* operon repression. According to Liu et al. (2007), when Cu concentration increases, one Cu(I) ion is bound to each CsoR monomer, leading to the dissociation of the protein from the operon and allowing transcription of these genes, including CtpV, the Cu^+^ transporter. In the case of *B. diazoefficiens*, it may be possible that expression of the *copA* gene, which encodes a putative Cu^+^ exporter, is derepressed in the Δ*csoR* strain grown under Cu-L, making the cytosolic Cu concentration even more limiting (Fig. 5). Previous results showed a strong inhibition of *nirK* and *norC* expression by Cu limitation in WT cells (Pacheco et al. 2022). The qRT-PCR results from the present work (Fig 4C and D) suggest that *csoR* deletion reinforces the repression of *nirK* and *nor* genes in response to Cu limitation. Moreover, the significant reduction of *norC* expression in Cu-S (Fig. 4D) could be explained by the increased fold-change value in *copA* expression (Fig. 4E) in Δ*csoR* compared to WT. Contrary to *norC* expression, *csoR* mutation did not affect *nirK* gene expression under Cu-S conditions (Fig. 4C). This observation suggests that *nor* gene expression might be more sensitive to the decline in Cu concentration inside the cell than *nirK* gene. Supporting this suggestion, Pacheco et al. (2022) demonstrated that Cu limitation had a greater negative effect on *norC* gene expression than on *nirK* expression by qRT-PCR analyses (33.25 versus 10.73 FC). Hence, the reduction in cytosolic Cu concentration in *csoR* Cu-S cells compared to WT cells (Fig. 3B) may preclude Δ*csoR* cells from reaching adequate *nor* expression. In order to investigate the possibility that CsoR could be a direct regulator of *nirK* or *norC* genes, we analyse the promoter sequences of those genes by using the FIMO (Find Individual Motif Occurrences) tool from the MEME suite. Only in the divergent promoter region between *copA* and *csoR*, two CsoR boxes were identified with *P*-values of 1.01 × 10^−8^ and 2.08 × 10^−8^ for each gene, respectively. However, putative CsoR boxes were not present in the *nirK* or *norC* promoter regions (data not shown).

Denitrification genes (especially *nor*) may be controlled by transcriptional factors able to detect transient Cu concentration shifts inside the cell. The ability of Cu to undergo redox changes, transiting from Cu(II) to Cu(I) and *vice versa*, makes Cu an ideal cofactor for enzymes catalysing electron transfer. The major redox state of Cu in bacterial cytoplasm is Cu(I) due to the low reduction potential maintained by low molecular-weight thiols (+0.15 V for Cu^+^ and −0.22 V for Cu^2+^) (Davis and O’Halloran 2008). However, Cu(I) is a strong soft metal and can attack and destroy iron–sulfur clusters thereby releasing iron, and consequently provoking oxidative stress (reviewed by Rensing and McDevitt 2013). Thus, we propose that Cu limitation could trigger transient changes in the cytosolic redox state that would be detected by the redox responsive RegSR system, as depicted in Fig. 5. In fact, previous findings show that, in addition to controlling *nifA*, RegR is also involved in *B. diazoefficiens* denitrification where it is required for induction of *nor* genes in response to anoxia and NO_3_^−^ (Torres et al. 2014). Torres et al. (2014) also demonstrated the capacity of RegR to bind *norC* promoter. Results from the present work allow us to propose that Cu-limiting conditions causes a cytosolic redox change that could be detected by the RegSR system, thus RegR would bind the *norC* promoter and block *nor* expression (Fig. 5A). This effect is strengthened in the Δ*csoR* strain (Fig. 5B). RegR might be also involved in the downregulation of *nirK* gene, but no evidences supporting this hypothesis have been reported yet.

To conclude, this study suggests that CsoR is not involved in *nirK* and *nor* gene regulation as a direct transcriptional factor, but it could influence indirectly the expression of these genes in response to the intracellular Cu concentration. Further investigation would be necessary in order to discern the potential connection between Cu homeostasis, the redox status of the cytoplasmic compartment, and regulation of denitrification.

## Supplementary Material

fnad084_Supplemental_Files
