# Supplementary material for: The copper-responsive regulator CsoR is indirectly involved in Bradyrhizobium diazoefficiens denitrification
Source: FEMS Microbiol Lett. 2023 Aug 12;370:fnad084. doi: 10.1093/femsle/fnad084 (PMC10457146; doi:10.1093/femsle/fnad084)
Supplement: fnad084_Supplemental_Files [file fnad084_supplemental_files.zip › Supplementary Material_FEMSLE-23-05-0144.R1.docx]

**Supplementary Material**

**Table S1.** Primers used in this study.

| **Primer** | **DNA sequence (5’→3’)** |
| --- | --- |
| *csoR*_Up_For_*Xba*I | ACATCTAGATCATGTCGGCGAGTTCGGGATTGG |
| *csoR*_Up_Rev_*Bam*HI | AAAGGATCCCTTGATGTCCTTGCGCATGCCGTC |
| *csoR*_Down_For_*Bam*HI | AAAGGATCCTGATCGGACGGGCGGAAAGGTAG |
| *csoR*_Down_Rev_*Eco*RI | AAAGAATTCTGCACTGGGCCGACAAGCAGAAG |
| pSRKC1_F | ATGCTTCCGGCTCGTATG |
| pK18_4 | CTGCGCAACTGTTGGGAAGG |
| *csoR*_IN_For | CTGAAGGACCACGTCGCCCATTG |
| Kan5 | ACAGGATGAGGATCGTTTCG |
| Kan6 | AATATCACGGGTAGCCAACG |

**Table S2.** Length, percentage (%) of identity and E-value between *B. diazoefficiens* 110*spc*4 CsoR protein sequence and the other selected sequences shown in Supplementary Figure S1. E-value exponents are indicated in parentheses; aa, amino acids.

| **Locus** | **Scientific name** | **Strain** | **Length (aa)** | **% identity** | **E value** |
| --- | --- | --- | --- | --- | --- |
| CP032617 | *B. diazoefficiens* | 110*spc*4 | 91 | - | - |
| BAC45966 | *B. diazoefficiens* | USDA 110 | 91 | 100 | 2e(−65) |
| WP_212372906.1 | *B. japonicum* | - | 91 | 97.80 | 5e(−63) |
| AMR68963.1 | *B. liaoningense* | CCNWSX0360 | 91 | 96.70 | 2e(−61) |
| WP_044409005.1 | *R. palustris* | - | 91 | 91.21 | 2e(−58) |
| MBA1946598.1 | *E. coli* | CE1762 | 75 | 81.58 | 9e(−40) |
| WP_085185770.1 | *B. subtilis* | CU1065 | 101 | 83.78 | 2e(−38) |
| TKW65881.1 | *P. denitrificans* | - | 93 | 45.24 | 1e(−25) |
| BAF53422 | *C. glutamicum* | R | 100 | 51.85 | 2e(−24) |
| HBO2457473.1 | *P. aeruginosa* | JARB-YO0198 | 97 | 43.08 | 2e(−19) |
| WP_191833845.1 | *P. fluorescens* | - | 95 | 42.42 | 5e(−17) |
| NP_215482 | *M. tuberculosis* | H37v | 119 | 36.67 | 1e(−14) |
| BAD71542 | *T. thermophilus* | HB8 | 94 | 35.06 | 2e(−12) |
| SFX13036.1 | *P. pantotrophus* | DSM-1403 | 90 | 33.33 | 2e(−10) |

**Supplementary Figure legends.**

**Figure S1.** Multiple amino acid sequence alignment of *B. diazoefficiens* 110*spc*4 CsoR (Fernández *et al*., 2019) against other selected CsoR homologs from *B. diazoefficiens* USDA 110 (Kaneko *et al*., 2002), *B. japonicum*, *B. liaoningense* (Liang *et al*., 2016), *R. palustris*, *E. coli*, *B. subtilis* (Smaldone and Helmann, 2007), *P. denitrificans*, *C. glutamicum* (Yukawa *et al*., 2007; Teramoto *et al*., 2012), *P. aeruginosa* (NCBI Pathogen Detection Project; Souvorov *et al*., 2018), *P. fluorescens*, *M. tuberculosis* (Camus *et al*., 2002; Liu *et al*., 2007; Lew *et al*., 2011), *T, thermophilus* (Sakamoto *et al*., 2010) and *P. pantotrophus*. Multiple alignment was carried out using Clustal Omega (<https://www.ebi.ac.uk/Tools/msa/clustalo/>). Highly conserved residues are shaded in light grey. Residues pointed with a black arrow are predicted to be essential for Cu(I) binding. Residues marked with a white arrow are predicted to be involved in protein-DNA interaction. The residues highlighted in grey without arrow are predicted to participate in the interaction between monomers. Locus designation, amino acid sequences and their references were retrieved from NCBI database (<https://www.ncbi.nlm.nih.gov/protein/>).

**Figure S2**. Structure of the *B. diazoefficiens* CsoR dimer model predicted by AlphaFold cartoon representation (neurosnap.ai, 15/07/2023). Each monomer is colored differently (green and orange), and labels indicate DNA- and Cu-binding domains, Cu(I) binding residues, and helix number.

**REFERENCES**

Camus J-C, Pryor MJ, Médigue C, Cole ST. Re-annotation of the genome sequence of *Mycobacterium tuberculosis* H37Rv. *Microbiology (Reading)* 2002;**148:**2967-73, <https://doi.org/10.1099/00221287-148-10-2967>.

Fernández N, Cabrera JJ, Varadarajan AR, Lutz S, Ledermann R, Rosckitzki B, Eberl L, Bedmar EJ, Fischer H-M, Pessi G, Ahrens CH, Mesa S. An Integrated Systems Approach Unveil New Aspects of Microoxia-Mediated Regulation in *Bradyrhizobium diazoefficiens*. *Front Microbiol* 2019;**10:**924, <https://doi.org/10.3389/fmicb.2019.00924>.

Kaneko T, Nakamura Y, Sato S, Minamisawa K, Uchiumi T, Sasamoto S *et al*. Complete genomic sequence of nitrogen-fixing symbiotic bacterium *Bradyrhizobium japonicum* USDA110. *DNA Res* 2002;**9:**189-97, <https://doi.org/10.1093/dnares/9.6.189>.

Lew JM, Kapopoulou A, Jones LM, Cole ST. TubercuList—10 years after. *Tuberculosis (Edinb)* 2011;**91:**1-7, https://doi.org/10.1016/j.tube.2010.09.008.

Liang J, Zhang M, Lu M, Li Z, Shen X, Chou M, Wei G. Functional characterization of a *csoR-cueA* divergon in *Bradyrhizobium liaoningense* CCNWSX0360, involved in copper, zinc and cadmium cotolerance. *Sci Rep* 2016;**6:**35155, <https://doi.org/10.1038/srep35155>.

Liu T, Ramesh A, Ma Z, Ward SK, Zhang L, George GN, Talaat AM, Sacchettini JC, Giedroc DP. CsoR is a novel *Mycobacterium tuberculosis* copper-sensing transcriptional regulator. *Nat Chem Biol* 2007;**3:**60-8, <https://doi.org/10.1038/nchembio844>.

Sakamoto K, Agari Y, Agari K, Kuramitsu S, Shinkai A. Structural and functional characterization of the transcriptional repressor CsoR from *Thermus thermophilus* HB8. *Microbiology* 2010;**156:**1993-2005, https://doi.org/10.1099/mic.0.037382-0.

Smaldone GT, Helmann JD. CsoR regulates the copper efflux operon *copZA* in *Bacillus subtilis*. *Microbiology* 2007;**153:**4123-8, <https://doi.org/10.1099/mic.0.2007/011742-0>.

Souvorov A, Agarwala R, Lipman DJ. SKESA: strategic k-mer extension for scrupulous assemblies. *Genome Biol* 2018;**19:**153, https://doi.org/10.1186/s13059-018-1540.z.

Teramoto H, Inui M, Yukawa H. *Corynebacterium glutamicum* CsoR Acts as a Transcriptional Repressor of Two Copper/Zinc-Inducible P_1B_-Type ATPase Operons. *Biosci Biotechnol Biochem* 2012;**76:**1952-8, <https://doi.org/10.1271/bbb.120437>.

Yukawa H, Omumasaba CA, Nonaka H, Kós P, Okai N, Suzuki N, Suda M, Tsuge Y, Watanabe J, Ikeda Y, Vertès AA, Inui M. Comparative analysis of the *Corynebacterium glutamicum* group and complete genome sequence of strain R. *Microbiology* 2007;**153:**1042-58, <https://doi.org/10.1099/mic.0.2006/003657-0>.
