# Supplementary figures and images for: The copper-responsive regulator CsoR is indirectly involved in Bradyrhizobium diazoefficiens denitrification
Source: FEMS Microbiol Lett. 2023 Aug 12;370:fnad084. doi: 10.1093/femsle/fnad084 (PMC10457146; doi:10.1093/femsle/fnad084)

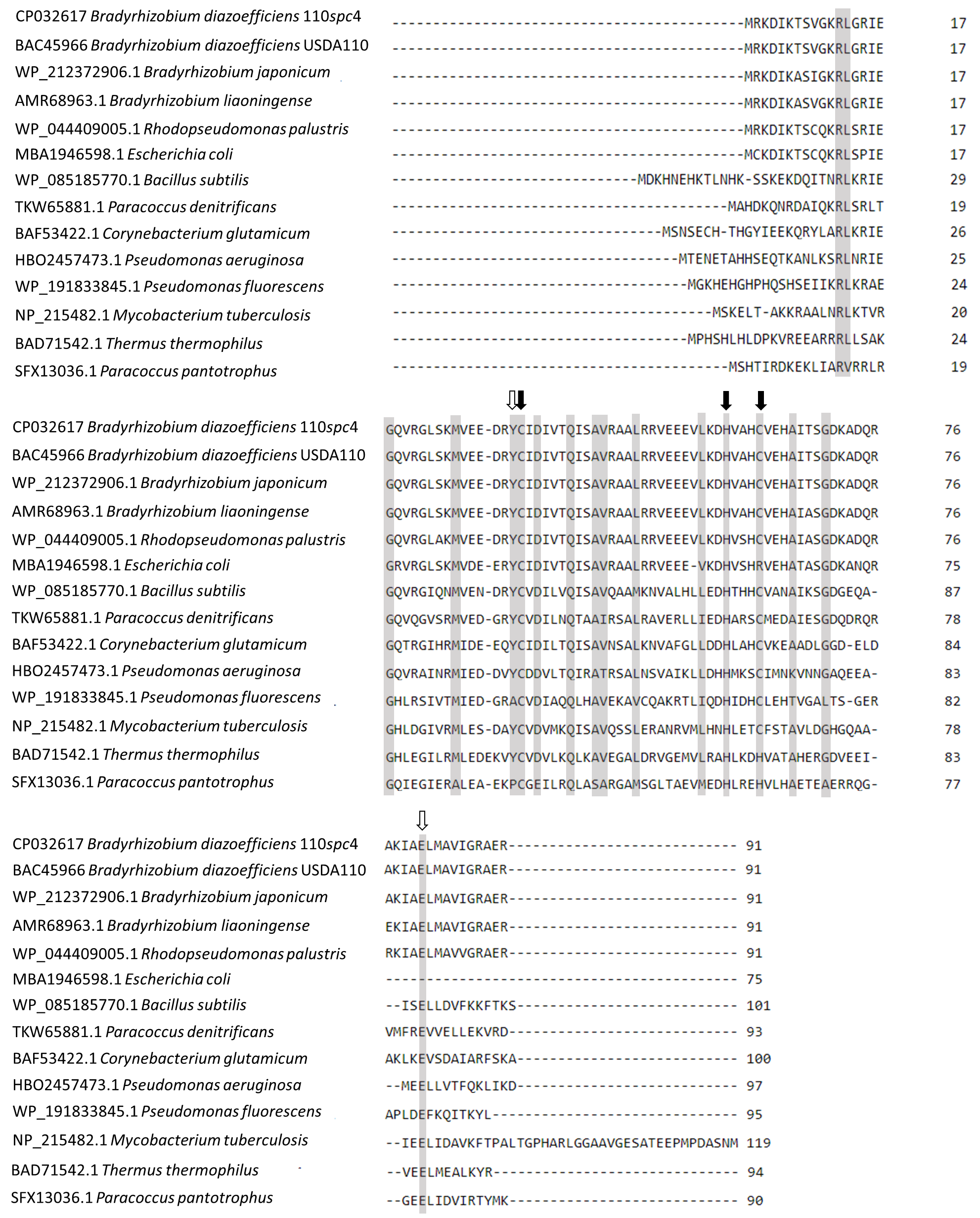

Supplement: fnad084_Supplemental_Files [file fnad084_supplemental_files.zip › Supplementary_Figure_S1.tif]

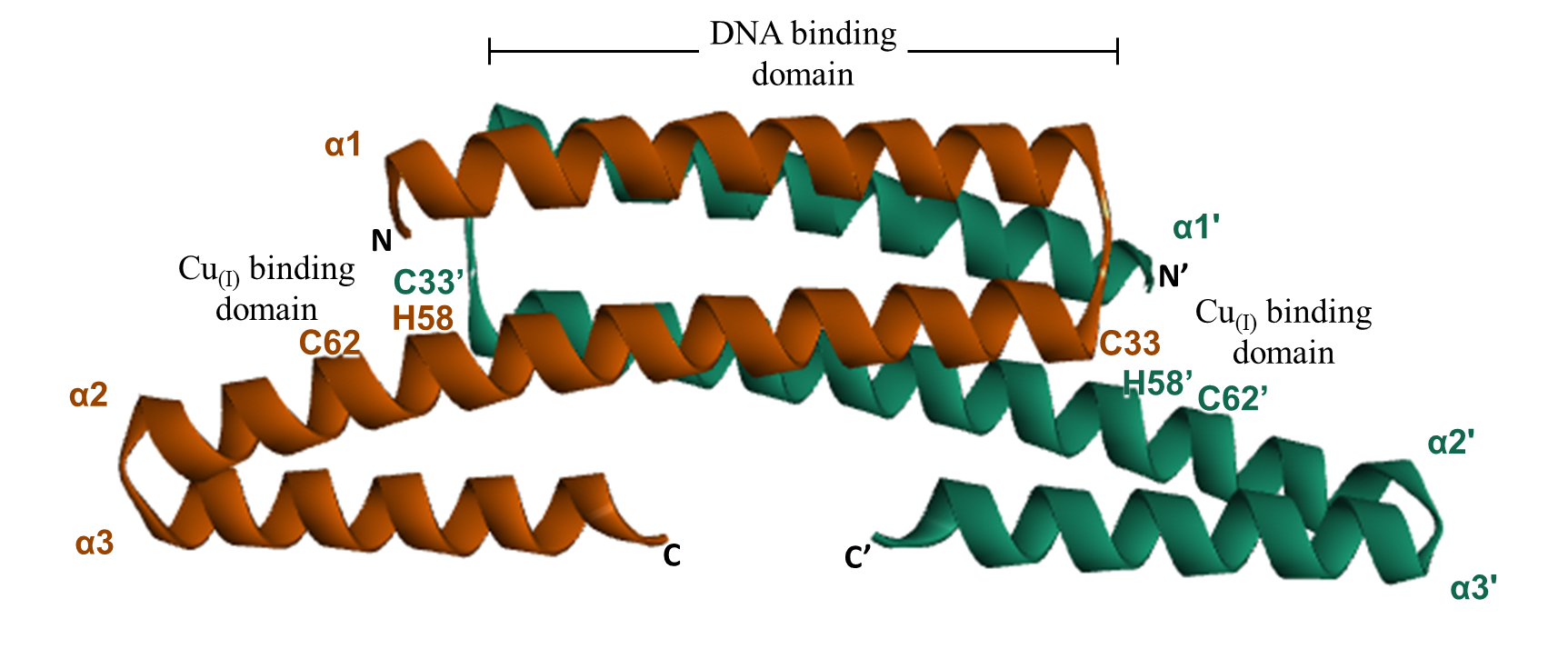

Supplement: fnad084_Supplemental_Files [file fnad084_supplemental_files.zip › Supplementary_Figure_S2.tif]
